# Shaping the Future of Chronic Kidney Disease Management in Spain: Insights from the CARABELA-CKD Initiative

**DOI:** 10.3390/jcm14051765

**Published:** 2025-03-06

**Authors:** Javier Escalada, Patricia de Sequera, Jesús Ignacio Diago, CARABELA-CKD Scientific Committee, Pedro Ruiz

**Affiliations:** 1 Spanish Society of Endocrinology and Nutrition (SEEN), 28001 Madrid, Spain; 2 Spanish Society of Nephrology (S.E.N.), 39008 Santander, Spain; 3 Medical Department, AstraZeneca Farmacéutica Spain, 28050 Madrid, Spain; jesusignacio.diago@astrazeneca.com; 4 Spanish Society of Quality of Care (SECA), 33003 Oviedo, Spain

**Keywords:** chronic kidney disease, clinical management, multidisciplinary approach, patient empowerment, healthcare quality improvement, standardized protocols

## Abstract

Chronic kidney disease (CKD) is a growing public health challenge in Spain, driven by population ageing and increasing risk factors. In this context, the CARABELA-CKD initiative brought together over 100 representatives from the CKD healthcare ecosystem in Spain to address this critical issue by identifying needs and improvement areas in the current management of CKD patients and proposing a comprehensive optimization of the Spanish CKD care process. This collaborative initiative involves leading scientific societies including the S.E.N. (Spanish Society of Nephrology), the SEEN (Spanish Society of Endocrinology and Nutrition), and the SECA (Spanish Society of Quality of Care), in partnership with AstraZeneca. CARABELA-CKD emphasizes data-driven decision-making and continuous quality improvement to identify and deliver innovative solutions that enhance patient care. Building on existing CKD care models, we performed an in-depth analysis of the current barriers in enhanced care and determined a series of healthcare quality indicators and areas for improvement. These areas encompass standardized protocols for care delivery, patient empowerment through education, and fostering collaboration among healthcare professionals and authorities. The CARABELA-CKD framework promotes a holistic, multidisciplinary approach, treating CKD management as a cross-functional process. As a result of this collaborative effort, a series of interventions are proposed, oriented to empower healthcare professionals to deliver personalized, high-quality care with the ultimate aim of improving patient outcomes and quality of life.

## 1. Introduction

Chronic kidney disease (CKD) is a major public health problem with increasing prevalence in most countries. In Spain, approximately 15.1% of the adult population are affected by this chronic disease, an incidence rate higher than in some European nations but comparable to the United States and Southern Europe [1]. CKD is a “silent epidemic”, particularly at the initial stages when symptoms have not appeared or are almost undetectable. This situation results in underdiagnosis or late diagnosis and an overall lack of public awareness [2]. The major risk factors for CKD include the progressive ageing of the population, hypertension, heart failure, obesity, and diabetes—all key contributors to the significant rise in the CKD burden in recent years that looks set to continue [1,3,4,5,6]. Recent data from the Spanish Dialysis and Transplant Registry (REDYT) indicate that DM2 and HTN remain the predominant causes of CKD in Spain, accounting for 25.2% and 13.5% of renal replacement therapy cases, respectively, and in line with global trends. This increasing burden and the associated mortality risk will potentially place CKD among the leading causes of death in Spain by the end of this century, when it is estimated that one in four people will have CKD [7]. Moreover, the demand for renal replacement therapies has risen by an overall 30% which translates into significant economic costs that consume 3% of the total Spanish health expenditure [8].

The growing impact of CKD on mortality and the advent of new diagnostic and therapeutic approaches have driven several international initiatives aimed at optimizing CKD care delivery at all stages, involving institutions such as the KDIGO (Kidney Disease: Improving Global Outcomes) Organization [9,10]. The International Society of Nephrology released a Comprehensive Care Plan in 2020 for patients with advanced CKD (ACKD) who require renal replacement therapy. These guidelines may provide a framework on which to base improvements over the next decade [11].

In Spain, several proposals at regional levels have addressed the comprehensive management of the CKD process, focusing mainly on the advanced stages of the disease [12]. The National Action Plan for Chronic Kidney Disease (2015–2020), developed by the Ministry of Health, Social Services and Equality, was released to improve the prevention, diagnosis, treatment and quality of life (QoL) of patients with CKD [8]. Moreover, the Spanish Society of Nephrology has developed a manual of quality standards for advanced CKD units in 2019 [3].

The National Action Plan represented a significant advance in the standardization of many clinical aspects of care and treatment for CKD patients. However, significant improvement areas remain an issue, as its implementation has been suboptimal, with low adherence to specific objectives and significant variations in outcome healthcare quality indicators across different regions. Moreover, the landscape of CKD has evolved in the last decade, and a new framework for kidney care in Spain is required [13]. There is an urgent need for a renewed focus on quality improvement strategies that promote the systematic use of healthcare quality indicators, continuous evaluation, and the establishment of well-coordinated clinical strategies that address CKD in a holistic way, with the overall goal of improving clinical outcomes impacting directly on patient survival, morbidity, QoL, and associated costs. These strategies should not only focus on preventing progression but also on ensuring early diagnosis and treatment [14,15].

In this context, the CARABELA-CKD initiative was developed as part of the CARABELA framework, built upon lean process reengineering principles [16]. The CARABELA-CKD initiative focuses on developing practical and implementable solutions that can be applied in a variety of settings, taking into account resource constraints, the organizational structures of the Spanish public healthcare system, and recognizing both patients and system diversities. CARABELA-CKD is headed by a multidisciplinary committee with the support of three scientific societies overseeing CKD management in Spain, namely the Spanish Society of Nephrology (S.E.N.), the Spanish Society of Endocrinology and Nutrition (SEEN), and the Spanish Society of Quality of Care (SECA), in partnership with AstraZeneca [17]. It aims to develop solutions tailored to the specific needs of different patient groups and regions. Using CARABELA methodology, healthcare professionals and experts in CKD care from various hospitals across Spain collaborated with five pilot hospitals and analyzed and discussed their findings in four regional and one national meeting to define improvement areas and potential solutions in CKD care management in Spain.

This paper is an integral part of the overall picture emerging from the CARABELA methodology [17] and specifically discloses the in-depth expert critical analysis of CKD management and its projected trajectory, delving into the established interventions in an attempt to overcome identified barriers and enhance the CKD care process.

## 2. Identifying Improvement Areas to Enhance CKD Management

As a result of the discussions and deliberations on the current complexities of CKD management, four major categories of improvement areas spanning diverse dimensions of healthcare provision were identified. These improvement areas, characterized by their intricate and multifaceted nature, demand a concerted attention and collaborative efforts to provide an integrated CKD care model of excellence.

### 2.1. Care Process Improvement Areas

Within the reality of clinical care, one notable obstacle is the scarcity of specialized nursing staff dedicated to CKD. Given the unique characteristics, chronic nature and risk of progression of this disease, trained nursing professionals are essential to ensure optimal patient care and service organization. Prior research underscored the indispensable role of nurses with nephrology expertise and emphasized the cost-effectiveness of implementing interventions led by a collaborative effort between nephrologists and specialized nurses to improve CKD patient care [18,19]. Nevertheless, the path to achieving this requires a concerted effort towards long-term, standardized training plans to raise awareness and equip healthcare professionals with the necessary skills and knowledge. This includes improving knowledge on the early introduction and appropriate use of nephroprotective agents, ensuring accessibility, and monitoring for potential side effects, as well as staying informed about emerging therapeutic options in clinical development. Moreover, the increasing clinical pressure and time constraints to which healthcare professionals are submitted, given the chronicity and high prevalence of CKD, limit their capacity for implementing new strategies and actions aimed at enhancing a more integrated care model.

During our deliberations, we identified another improvement area: the frequent turnover of healthcare personnel. Despite efforts to provide ongoing training on CKD management and patient referral protocols, this dynamic environment hinders the steady transfer of knowledge.

The lack of clearly defined and standardized diagnostic and referral criteria and protocols adds another layer of complexity to CKD management. Uniform and updated protocols ensure optimized care processes, reduces waiting times, and encourages the continuity of patients care.

Moreover, the absence of communication and coordination plans makes it difficult to guarantee a healthcare continuum. In this context, national and international initiatives focused efforts to establish multidisciplinary care unit models of care for CKD aiming to address this improvement area and fulfil a comprehensive care and improve the QoL and survival of CKD patients [3,20,21]. These patients, particularly in initial disease stages, are often followed up by primary care services, so to avoid redundancies and potential inefficiencies impacting patients, fluent communication and coordination between primary care, nephrology, endocrinology, nutrition, and other specialties are vital [22].

On a broader level, the lack of tools to measure patient experience impedes our ability to assess the efficacy of healthcare services, as it inhibits the comprehensive evaluation of clinic interactions, diagnostic protocols, and overall healthcare delivery. Recording data on patients’ feedback regarding their clinic visits, diagnostic procedures, and overall experiences helps identify patients who maintain stable disease conditions over time, providing evidence for resource optimization and awareness campaigns.

### 2.2. Social Improvement Areas

Social improvement areas are multifaceted, spanning patient characteristics, disease progression, age demographics, individual circumstances, and expectations. The lack of structured patient education initiatives exacerbates the issue, particularly given the chronicity of CKD. Implementing tailored education programmes becomes imperative to empower patients in recognizing disease progression, identifying warning signs, and adopting proactive self-care strategies and healthy lifestyle behaviours [23]. Addressing these social improvement areas is paramount to advancing CKD management practices and optimizing patient outcomes.

### 2.3. Technological Improvement Areas

The integration of innovative technologies into daily clinical practice poses a significant yet exciting improvement area for clinicians. Clinical information systems play a pivotal role in delivering evidence-based, patient-centred care by enhancing data accuracy and accessibility, minimizing medical errors, guiding decision-making processes, and ultimately improve the quality and efficiency of healthcare delivery. One key obstacle lies in the low compatibility of these advancements with existing information systems [24,25]. This integration relies not only on technological expertise but also on enhancing communication channels among diverse healthcare specialties, promoting multidisciplinary collaboration, and ultimately, delivering more comprehensive care to CKD patients. However, navigating the complexities of modern information systems presents its own set of improvement areas. Tailored training programmes led by seasoned experts are indispensable for clinicians to fully exploit the potential of these systems [26]. Such initiatives not only serve to familiarize healthcare professionals with the functionalities and complexities of these systems but also empower them by optimizing daily operations and thereby maximizing efficiency and patient outcomes. The predictive ability of artificial intelligence models is essential in healthcare management, as it supports the early detection of individuals at risk for kidney disease and allows for timely and effective interventions [27].

### 2.4. Organizational and Economic Improvement Areas

Beyond the clinical and social improvement areas lie significant organizational and economic barriers that demand attention and require strategic interventions at various levels of healthcare centre operation and regulation [28]. Insufficient resources, both human and infrastructural, pose a critical barrier to the effective implementation of action plans aimed at CKD improvement and impede the evolution of healthcare centres, exacerbating issues such as prolonged waiting lists and decreased system efficiency.

The engagement of medical leadership and administrative bodies is principal to address these improvement areas. By conducting comprehensive data collection, centres can identify specific needs and promote the implementation of improvements, thus enhancing their positioning at both regional and national levels.

Financial limitations and budgetary constraints further complicate the sustainability of the healthcare system, particularly in the face of population ageing and disease chronicity. Thus, the implementation of innovative funding models advocating for equitable resource allocation and standardized care approaches is imperative.

Active involvement of administrative bodies in public awareness campaigns and disease prevention initiatives is crucial given the widespread lack of knowledge of CKD among the general population. Additionally, improvement areas in talent acquisition and retention underscore the importance of robust policies aimed at retaining and attracting healthcare professionals. High turnover rates and convoluted hiring processes hinder efforts to respond to patient needs, improve health outcomes, and implement vital disease prevention strategies such as patient education.

## 3. Solutions to the Current CKD Care Models

**Figure 1:**
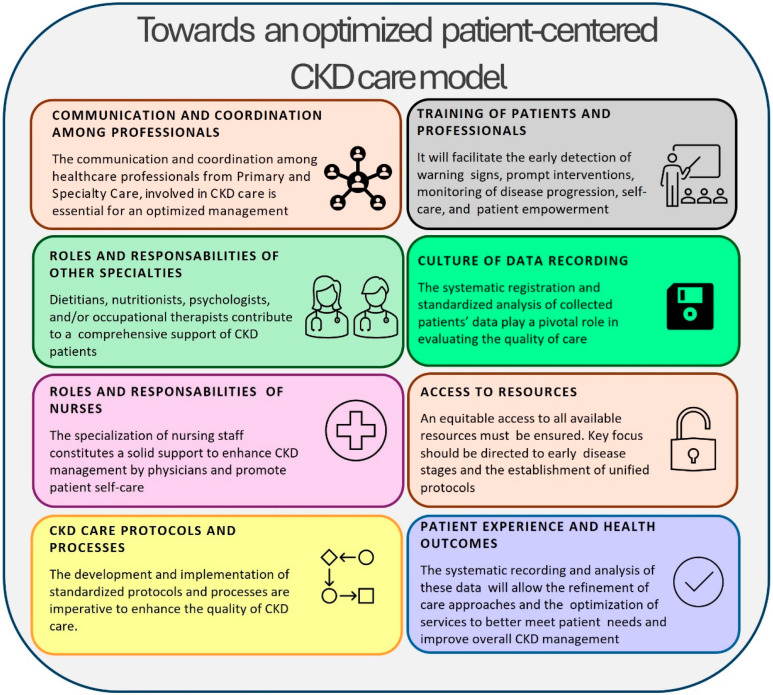
Potential solutions in current CKD management models identified during the development of CKD-CARABELA initiative.

The thorough analysis of current CKD care models operating in Spain led to the identification of potential solutions (Figure 1) and to the development of a set of healthcare quality indicators during the National Conference held as part of the CARABELA-CKD initiative development. A total of eight potential solutions were further refined at regional levels, leading to the definition of seventeen specific lines of action to address these needs [29].

### 3.1. Communication and Coordination Among Healthcare Professionals Involved in CKD Care Is Crucial

The seamless collaboration and effective communication among healthcare professionals, encompassing both Primary Care (PC) and Hospital Care (HC), is a cornerstone in optimizing CKD management [22]. It is clear to clinicians immersed in the daily improvement areas of CKD care delivery that a cohesive approach, underpinned by robust coordination mechanisms, is essential for ensuring a holistic patient support and care continuity. Based on our collective insights and deliberations, certain lines of action emerged as pivotal in advancing CKD care delivery in Spain.

One such strategy relies on the establishment of a dedicated case management role to facilitate smooth intra- and extra-hospital transitions for CKD patients navigating between PC and HC settings. Such a figure may provide improved multidisciplinary communication and truly integrated CKD care by proactively assessing patient care plans, addressing risk factors, and providing education on CKD and other support as needed [21,30,31].

Our reflections also underscored the importance of multidisciplinary clinics encompassing Diabetic Nephropathy, Cardiorenal, and Vasculo-renal consultations. By recognizing the intricate interplay between CKD and various comorbidities associated with other conditions (mainly diabetes and cardiovascular diseases), this integral approach will ensure that patients with multiple conditions receive comprehensive care tailored to their individual needs, thereby optimizing their follow-up and treatment outcomes and enhancing their QoL.

Given the primordial role of a multidisciplinary approach, we recommend the implementations of particular interventions aimed at enhancing this holistic vision:

- The development, standardization, and implementation of effective communication pathways between primary and hospital care settings, as well as among various hospital specialties involved in the care process. This is essential to facilitate a seamless information exchange throughout the continuum of care, thus ensuring comprehensive support for CKD patients.
- The evolution of CKD clinics towards a multidisciplinary comprehensive care model might be a pivotal strategy to enable the integration of diverse professional profiles and key specialties in the provision of integral CKD care. By fostering collaboration and communication among healthcare professionals, this model would facilitate an equative access to resources and enhance overall care quality.
- The creation of an integrative and unifying professional profile with a cross-sectional coordination role, decision-making competencies and autonomy is key to achieve real coordination and contact between healthcare professionals.
- Lastly, the promotion of the case manager role is a critical step in facilitating a smooth transition for CKD patients between intra- and extra-hospital settings. A designated case manager may assume responsibility for the coordination and continuity of care, ultimately improving each CKD patients’ journey.

In this respect, it is important to note the reported impact of the introduction of a multidisciplinary care and proactive case management model in the early improvement of the quality of care and prevention of disease progression among CKD patients [21,30,31].

### 3.2. The Roles and Responsibilities of Other Specialties Contribute to the Comprehensive Support of CKD Patients

This issue is of particular importance in the management of CKD patients who often present with multiple comorbidities requiring prior monitoring by various professionals. Addressing the complex needs of CKD patients, particularly those in advanced stages, and their families, requires comprehensive support in several aspects. In this context, roles and responsibilities between PC and HC can overlap significantly [32].

An important line of action identified during the deliberations was the inclusion of nutritional, psychological, and occupational therapy specialties in the CKD care process [33,34,35]. Dietary management is crucial to prevent disease progression. In addition, the valuable contribution of occupational therapists and psychologists is instrumental in helping patients to normalize their condition and learn how to deal with the physical and emotional improvement areas associated with their condition.

Having determined the importance of involving diverse areas of specialization for an enhanced CKD care process, we propose the following interventions:

- Development of standardized action protocols for the treatment and follow-up of CKD patients, which incorporate the expertise of dietitians, nutritionists, psychologists, and/or occupational therapists, with the aim of providing holistic support to patients, addressing both the physical and psychological dimensions of their journey.
- Establishment of periodic, structured, scheduled, and mandatory training sessions within each care service to update the knowledge of physicians involved in CKD management and facilitate the exchange of experiences between PC and HC, fostering interdisciplinary collaboration.
- Implementation of plans to promote the adoption of technological tools that facilitate proactive patient management and alert physicians to patients requiring specific and immediate attention.

### 3.3. The Important Roles and Responsibilities of Nurses Specialized in CKD Management

A pivotal aspect in CKD care, although often overlooked, is the specialization of nursing staff who offer a solid support to enhance disease management by physicians and promote patient self-care. By investing in nursing specialization, significant benefits can be achieved by reducing clinic visits and waiting times, preventing unnecessary hospital admissions, enhancing patient satisfaction, and ultimately optimizing healthcare costs [36]. Prior studies showed that cooperation between specialist nurses and primary healthcare professionals improved the risk management of CKD patients while delaying disease progression [37].

Nursing personnel play a primary role in educating and empowering CKD patients, encouraging their active involvement and slowing disease progression. Patient education should encompass dietary guidance, promotion of healthy lifestyle habits (such as moderate physical activities adapted to individual circumstances), recognition of warning signs indicating progressive disease, and care protocols tailored to individual patient needs.

It is, then, essential that CKD specialized nursing professionals are exclusively dedicated to facilitating focused and efficient CKD care delivery.

Regarding nursing roles and functions, important action lines have been identified, including primarily the incorporation of dedicated and specialized nursing staff in CKD/ACKD consultations. Although some nursing personnel do specialize in CKD/ACKD, their focus on CKD patients may be diluted. Establishing dedicated nursing roles enhances care coordination and promotes patient-centric care experiences, facilitating the collection and evaluation of Patient-Reported Outcome Measures (PROMs) and Patient-Reported Experience Measures (PREMs). This valuable information provides crucial data to healthcare professionals at the time of confirming decisions. This approach will drive improvements, draw insights from other specialties or medical facilities, and trigger cooperation and change.

To this end, we recommend:

- The promotion of specialization programmes for nursing personnel within healthcare services, who may gain accreditation in CKD management from relevant official institutions involved in disease management, such as Scientific Societies.
- The development of robust monitoring protocols that reinforce the role and function of nursing in terms of education, training, patient monitoring, and coordination. These protocols will serve as a framework for both patients and clinicians, ensuring standardized care practices.
- The establishment of dedicated nursing consultation spaces equipped with the necessary technology and equipment for patient monitoring and follow-up, optimizing the delivery of CKD care services.

### 3.4. The Refinement of CKD Care Protocols and Processes Is the Path to Improved Clinical Outcomes

CKD care features multifaceted improvement areas due to its inherent comorbidities and the complex interplay of intra- and extra-hospital processes. The development and implementation of standardized protocols that drive improvement areas are imperative to streamline referral criteria and enhance the quality of CKD care. Addressing the existing improvement areas in current healthcare systems requires the creation of guidelines outlining procedures for both hospital and primary care settings, thereby promoting standardized practices among healthcare professionals.

The cornerstone of effective CKD management rests on the establishment of robust referral protocols. Given the prevalence of CKD and associated healthcare costs, it is vital to minimize unnecessary referrals [38]. Delays in patient referrals due to ambiguous or outdated criteria also lead to bottlenecks in the diagnostic process. Therefore, the adoption of clear and current referral protocols is paramount to achieve early CKD diagnosis and mitigate the burden of waiting lists. The creation and implementation of referral protocols to Nephrology or Day Hospitals from Emergency Departments, PC, and other hospital specialties and the promotion of multidisciplinary collaboration will enhance the quality and efficiency of the CKD care process, while offering an important improvement in patient experience. The standardized protocolization of care pathways for chronic patients that also prioritize the creation of multidisciplinary working groups will ensure comprehensive and cohesive patient management across specialties.

An important strategy considered in all value-based healthcare models is the identification of specific chronic patient groups who will be the focus of multidisciplinary teams working on developing a patient-centred care [39].

Given these crucial action lines, our recommendations include:

- The definition and dissemination of comprehensive patient care protocols integrating insights from different healthcare professionals and care levels. This approach will ensure a holistic and patient-centred care continuum, promoting optimal CKD management.
- The establishment of rapid pathways and protocols ensuring their proper utilization for chronic patients care. Timely intervention is essential in mitigating disease progression and improving patient outcomes.

### 3.5. The Evolution of the CKD Management Model Pivots on Prioritizing the Education of Patients and Training of Professionals

The importance of enhancing training for healthcare professionals involved in CKD management and education for patients themselves cannot be overstated. Indeed, education is a critical component in engaging patients with kidney disease and opens a pathway towards self-management and patient-centred care which should frame every physician–patient interaction.

All specialties involved in CKD management should undergo continuous training to recognize early warning signs in diagnostic results or patient symptoms. The screening of high-risk individuals, communication, and involvement of CKD patients in their own healthcare will give clinicians the tools they need to address many patient-level barriers and achieve an effective education and shared decision-making [40]. Such initiatives are crucial for the early detection of warning signs prompting straightforward interventions to mitigate harm [41], monitoring of disease progression, self-care, and patient empowerment. These measures will also ensure appropriate patient referral to HC, thereby optimizing patient pathways across all specialties and throughout the care continuum [42]. Moreover, all these actions are associated with better outcomes [43].

In this regard, specific action lines were identified during CARABELA-CKD meetings. Along with early disease detection, a successful model of CKD care should incorporate the timely and efficient referral of patients to HC, as the large volume of conditions and patients managed by each professional involved in CKD care can lead to inefficiencies in these processes. However, despite guideline recommendations (including KDIGO guidelines), the efficiency in nephrology referrals vary between different healthcare models [44,45]. Thus, the definition of structured training sessions for specialists involved in CKD management by Nephrology departments can play a pivotal role in promoting standardized referral criteria.

In addition, the high patient demand in the current healthcare system means that professionals are facing increasing time constraints when adequately addressing the needs of well-informed patients. In this regard, the use of educational resources customized for individuals with limited health literacy can enhance disease awareness and promote better self-management and QoL among CKD patients [46,47,48]. We believe that the creation and implementation of gamified and dynamic health education programmes (focusing on lifestyle and self-care), facilitated by technology, must surely enhance patient engagement and autonomy in their learning process, ultimately improving treatment adherence. From the clinician’s perspective, effective training and education supports meaningful shared decision-making.

Considering the crucial importance of increasing education and awareness on CKD and its preventive measures, we suggest the following measures focused on the identified action lines:

- Develop tailored training plans for healthcare professionals involved in CKD management and treatment throughout the care process. These plans should cater to the specific needs of professionals, enhancing their understanding of CKD and enabling a more comprehensive approach to disease management.
- Create patient education programmes adapted to individual needs and digital literacy, incorporating technological elements that facilitate patient education on CKD management and self-care (e.g., gamification tools). It is also important to maintain a continuous supply of valuable materials in physical formats for non-digital patients, ensuring their inclusion in the educational initiatives.

### 3.6. The Promotion of a Culture Focused on Data Recording Is a Key Driver of Enhanced CKD Care

The systematic recording and standardized analysis of data play a pivotal role in evaluating the quality of care provided to CKD patients [49]. The advent of comprehensive databases housing standardized healthcare quality indicators marks a new era of personalized medicine and empowers clinicians to identify areas of improvement within the care process and establish collaborative networks among healthcare professionals. The cultural attitude towards data registration and exploitation within healthcare institutions has undergone a paradigm shift and emerging technologies have facilitated the exponential generation of data. This underscores the urgent need to develop innovative financing models and initiatives that ensure the sustainability of CKD management models which proficiently handle patient data in compliance with data protection regulations.

Robust data registration and measurement of healthcare quality indicators across diverse parameters are instrumental in identifying areas of improvement and best practices within healthcare facilities [49,50,51]. Thus, we identified as a crucial action line the identification of key healthcare quality indicators for CKD management to enhance awareness and promote a culture of data registration and exploitation. The active involvement of authorities, scientific societies, and healthcare leadership is essential for defining and periodically evaluating these healthcare quality indicators, establishing benchmarks, and monitoring the evolution of service or facilities across various dimensions. Another line of action focuses on awareness programmes for professionals, which emphasize the significance of diagnostic coding. Consistent and standardized coding of patient diagnoses enables the continuous registration of established parameters and the subsequent data retrieval for potential studies and ensures precise patient monitoring. In this respect, communication plans are crucial to raise awareness about the importance of this initiative.

We recommend a series of proactive measures that will contribute to the culture of data-driven decision-making and ultimately improve CKD patient outcomes:

- Incorporation of technological profiles or improvement of existing profiles in utilizing data registration tools/platforms and effectively exploiting and evaluating generated information.
- Development of protocols that not only enhance data dissemination among healthcare professionals across different care levels to improve CKD management but also ensure the security and protection of data generated throughout the care process by defining data ownership and storage locations both in intra- and extra-hospital settings (e.g., data from smart devices, patient associations, etc.).
- Design of dashboards that enable the efficient exploitation and visualization of valuable information generated within the hospital environment.

### 3.7. The Evolution of CKD Care Must Encompass Equity in Access to Resources

The evolution of CKD management towards a comprehensive and integrated approach must ensure equitable access to all available resources, regardless of the responsible department or referring centre. In this respect, the availability of resources from healthcare departments and support services has been identified as an urgent need, along with the implication and motivation of departments to implement solutions in their care processes. The key focus should be on early disease stages and unified protocols for sample collection, data gathering, and determining variables across healthcare facilities.

In terms of resource accessibility, we identified various targeted strategies. Hospital care is crucial for patients in advanced CKD stages. Thus, expanding consultation services dedicated to CKD patients can optimize care, alleviate waiting times, and enable consistent and specialized monitoring. Moreover, creating or enhancing a versatile Day Hospital model that integrates all relevant specialties in CKD management will provide patients with a more comprehensive hospital care experience.

Given the multidisciplinary nature of CKD management, the implementation and/or unification of an official and effective communication channel between PC and HC with the support of information systems could also enable the coordination and optimization of diagnostic processes, facilitating the receipt of diagnostic tests and instructions from the responsible physician for process optimization.

Lastly, the development of home admission models is crucial, particularly considering that CKD is often associated with advanced age. This approach might mitigate the impact of hospital transfers on the QoL of both the elderly CKD patients who experience reduced autonomy due to the disease and their caregivers. Thus, the creation of home admission models is invaluable for optimizing the management of these CKD populations and enhancing their experience.

To address these proposed strategies, we recommend:

- The establishment of expert networks operating within a unified care framework. Such networks facilitate equitable patient access to the latest available technological advancements in CKD management. They also foster collaboration between HC and PC by creating a common resource structure between both settings and shared access to educational and technological resources, ultimately enhancing the quality of care provided to CKD patients.

### 3.8. The Importance of Measuring Health Outcomes and Patient Experience

As clinicians deeply involved in managing CKD, we recognize the significance of patient engagement. Insights into the wealth of information they possess, their personal experiences and their health outcomes are invaluable for refining our approach to care. By systematically recording and analyzing these data, we can refine our care approaches and optimize services to better meet patient needs and improve overall management.

Our reflections based on our expertise in managing CKD in real-world practice led us to identify potential lines of action. First, the standardization and development of tools for collecting and evaluating PROMs and PREMs from CKD patients are essential to assess patient satisfaction. PROMs are increasingly recognized as valuable tools to evaluate the effectiveness of patient-centred CKD interventions [52,53], as understanding patients’ perspectives on health outcomes and quality of care is essential for enhancing healthcare delivery. The collection and analyses of PROMs and PREMs and the implementation of necessary adjustments in medical interventions will notably enhance patient satisfaction.

We also advocate for the implementation of health promotion and CKD prevention programmes, particularly considering that despite its high prevalence, CKD remains relatively underrecognized. Primary CKD prevention can only be accomplished by raising the awareness of CKD among healthcare professionals, patients and authorities as a major public health problem, and providing information about common associated conditions, preventive measures, detection methods, potential complications, and implications [54,55]. In this regard, collaboration with influential public institutions is crucial.

Furthermore, we support the initiation in PC of patient education programmes to create expert patient groups and facilitate patient empowerment. Patient empowerment not only fosters social connections and creates a supportive community among patients but also allows them to empathize and potentially improve disease prevention, providing awareness and knowledge, and to participate in shared decision-making with clinicians, thus facilitating self-management [56]. Also, it is important to consider that, given the chronic nature of the disease, the optimization of patients’ quality of life and their journey through the healthcare system requires an adequately trained nursing staff.

To translate these insights into action, we propose:

- The creation of simple and concise questionnaires with defined parameters to efficiently capture and extract patient information.
- The development and/or updating of information systems capable of quickly and efficiently extracting and analyzing the data, thus facilitating prompt decision-making and service optimization.

## 4. Conclusions

CARABELA-CKD has reshaped our approach as clinicians to CKD management by providing an innovative and distinctive vision from prior initiatives, due to its foundation in real-world clinical practice and the expertise of a diverse group of experts, ensuring that all findings and recommendations are grounded in the realities of everyday care and directly addressing the needs of patients and healthcare professionals. Given the high prevalence and increasing burden of CKD in Spain, this initiative also underscores the urgent need for structured early detection strategies and population-level interventions to mitigate disease progression and reduce its impact on healthcare systems. This transformative process emphasizes the importance of continuous improvement and cross-functional collaboration among healthcare professionals, advocating for a holistic multidisciplinary approach, a culture of data-driven decision-making and patient-centred care. Through the development of innovative strategies and partnerships with stakeholders across the healthcare ecosystem, we strive to create sustainable improvements and develop tailored solutions that cater to the diverse needs of our patients, adding value to their experience.

The identification of key improvement areas in the current CKD management process which span diverse dimensions has underscored the complexity of CKD management. In this scenario, multidisciplinary collaboration, standardized protocols, and patient empowerment are key elements for a collective effort towards enhancing the current CKD care process. The ultimate goal is to deliver personalized, high-quality care and empower patients to take an active role, thus elevating the standard of CKD care in Spain. By prioritizing patient engagement, standardizing data collection methods, and addressing preventive efforts, CKD care will surely benefit, and patient outcomes will ultimately improve. Addressing these challenges with a broader public health perspective will ensure a more sustainable and effective approach to CKD management at both individual and population levels.

## Data Availability

The original data supporting the conclusions of this article are openly available in [29] at doi.org/10.1016/j.nefro.2024.11.001.

## Abbreviations

The following abbreviations are used in this manuscript:

ACKD	Advanced chronic kidney disease
CKD	Chronic kidney disease
HC	Hospital care
PC	Primary care
PREMs	Patient-Reported Experience Measures
PROMs	Patient-Reported Outcome Measures

## Appendix A

The CARABELA-CKD Scientific Committee consists of the following members: Borja Quiroga (Sociedad Española de Nefrología, S.E.N.), Javier Escalada (Sociedad Española de Endocrinología y Nutrición, SEEN), Juan José Gorgojo (SEEN), Manuel Pérez Maraver (SEEN), Mercedes Salgueira (S.E.N.), Patricia de Sequera (S.E.N.), Pedro Ruiz (Sociedad Española de Calidad Asistencial, SECA), Alberto Prado Dominguez (Departamento médico, AstraZeneca Farmacéutica Spain), Jesús Ignacio Diago (Departamento médico, AstraZeneca Farmacéutica Spain), and Lucía Regadera (Departamento médico, AstraZeneca Farmacéutica Spain).
